# Multi-Country Estimate of Different Manifestations of Aspergillosis in Cystic Fibrosis

**DOI:** 10.1371/journal.pone.0098502

**Published:** 2014-06-10

**Authors:** Joanne Armstead, Julie Morris, David W. Denning

**Affiliations:** National Aspergillosis Centre, University Hospital South Manchester, The University of Manchester and Manchester Academic Health Science Centre, Manchester, United Kingdom; University of Tübingen, Germany

## Abstract

*Aspergillus spp*. can lead to allergic bronchopulmonary aspergillosis (ABPA), *Aspergillus* sensitisation and *Aspergillus* bronchitis in CF. The relative frequencies of these entities have recently been ascertained in a large UK adult CF cohort. We have used this data to estimate the burden of aspergillosis and ABPA cases in adult CF patients in 30 countries reporting CF. National and international CF registry data was accessed and assessed for completeness and age distribution. Published proportions of ABPA (17.7%), *Aspergillus* sensitisation (14.6%) and *Aspergillus* bronchitis (30%) in CF were applied to those >18 years and compared with notified ABPA cases. Of the 76,201 estimated CF patients worldwide (not including India), 37,714 were >18 years. The proportion of adults to children varied from 63% in Norway to 20% in Brazil. ABPA caseload in adults is anticipated to be 6,675 cases of which only 2,221 cases (33%) are currently recorded, indicating substantial underdiagnosis. The ABPA diagnosis rate compared with estimated rates varies by country from 101% (France) to 14.5% (Greece), although genetic variation could account for genuine differences compared with the UK. *Aspergillus* bronchitis is not currently recognised or recorded in CF registries but there are an anticipated 10,988 adult cases. *Aspergillus* sensitisation, associated with increased bronchiectasis and reduced FEV1, affects an anticipated 5,506 patients without ABPA or *Aspergillus* bronchitis. Together ABPA and *Aspergillus* bronchitis are estimated to affect 17,989 adults, 47.7% of the adult CF population. ABPA also occurs in children and teenagers and 984 cases were documented in registries. Diagnosed ABPA rates by age were available for the ECFS registry, USA, UK, Ireland, Belgium and Netherlands. The rate was <1% under 4 years, and increased throughout childhood and adolescence, with marked variation between countries. Newly published diagnostic criteria and methods should facilitate better recognition of aspergillosis in CF, allowing better CF disease control.

## Introduction

Cystic fibrosis (CF) is a recessive genetic disorder caused by the mutation of the cystic fibrosis trans membrane conductance regulator (CFTR) gene [1]. The CFTR protein plays a vital role in transporting salt and water across the surface of epithelia [2]. The abnormal function of this protein affects many organs of the body, especially the lung epithelia whereby the production of thick, sticky mucous often results in chronic lung infection and inflammation[2]. *Aspergillus* is a fungus present in both indoor and outdoor environments [3]; however it only tends to cause infection within susceptible individuals, such as those with CF.

*Aspergillus fumigatus* is the most common species of *Aspergillus* that causes morbidity in patients with CF. Colonisation of this fungus can manifest in various ways such as IgE-mediated sensitisation and allergic bronchopulmonary aspergillosis (ABPA). Varying prevalence of ABPA has frequently been reported since it was first diagnosed in 1965 by Mearns et al [4]. The Epidemiologic Registry of CF data involving 12,447 adult and children CF patients gathered from 224 CF centres within nine European countries was analysed and suggested 7.8% of patients had ABPA [5]. This figure varied between the countries, ranging from 2.1% in Sweden to 13.6% in Belgium. ABPA was also found to be less prevalent in patients below the age of 6 [5]. A similar study involving 14,210 patients from the Epidemiologic Study of CF reported a much lower prevalence of 2% [6]. Other smaller studies have found higher rates of up to 15% patients being diagnosed with ABPA [5], [7], [8]. There are increased ABPA rates within adolescent [6] and adult populations [9]–[12]. ABPA is often diagnosed late; on occasions as long as 10 years after presentation [13]. Generally the disease is under-diagnosed globally, and an estimated 4.8 million adults with asthma are affected [14]. Many in asthma cases are still misdiagnosed as pulmonary tuberculosis, [15] with some studies suggesting 17–50% of ABPA cases are misdiagnosed [16]. Early diagnosis would enable better management of aspergillosis and probably an improved prognosis in CF patients.

The proportion of CF patients with *A. fumigatus* colonisation in respiratory secretions ranges from 6% to 58% [16]–[18]. A retrospective cohort study involving 259 patients found 61 (24%) were at least intermittently colonized with *A. fumigatus* between 2002 and 2007 [17]. Culture methods vary substantially between centres, and culture is an insensitive means of determining colonisation [19]. A colonised patient may or may not have ABPA or another manifestation of aspergillosis.

Baxter et al. recently developed a novel diagnostic classification system of aspergillosis in adult CF patients of *Aspergillus* hypersensitivity and colonisation beyond ABPA [19]. In this study sputum samples were collected from 130 patients not receiving azole therapy and analysed to detect *Aspergillus* using two new methods; real-time PCR (RT-PCR) and galactomannan (GM) antigen. These tests were combined with the standard serologic analysis to create a new classification system of aspergillosis in CF [19]. Latent class analysis of these samples demonstrated three distinct classes of aspergillosis in adults with CF and one non-diseased class. 49 patients (37.7%) were in class 1 which represents non-diseased CF patients with or without *Aspergillus* in sputum but negative GM and no immunologic response. 23 patients (17.7%) had ABPA and were placed in class 2 whereby the patient had all high immunologic markers, positive RT-PCR and positive GM. 19 patients (14.6%) were *Aspergillus* IgE-sensitised in class 3 with or without Aspergillus in sputum but negative *A. fumigatus* IgG and negative GM. Finally class 4 involved 39 patients (30.0%) with *Aspergillus* bronchitis (AB) who demonstrated negative IgE markers but positive *A. fumigatus* IgG, RT-PCR and GM. These data clearly need replication in other centres, but serve as a basis for an initial estimate of CF-associated aspergillosis burden.

This quantitative evidence base on which to diagnose ABPA, AB and AS is an improvement on the previous criteria for diagnosing ABPA. We therefore set out to estimate the prevalence of different forms of aspergillosis in CF in as many countries as possible based on the recent publication of the different phenotypes of aspergillosis in CF. Where pediatric data on ABPA frequency was available, we have included this, but not modelled *Aspergillus* sensitisation or bronchitis.

## Methods

### Literature search for ABPA rates

A literature search on published data on ABPA and *Aspergillus* colonisation frequency in CF was done using Medline, Embase, Scopus, Ovid and Google scholar in July 2013. Searches for the prevalence of ABPA in CF in different countries were also conducted. The quality of the study for epidemiologic purposes was evaluated by assessing the diagnostic criteria for ABPA and *Aspergillus* sensitisation, and separately for *Aspergillus* colonisation. In addition particular attention was paid to the denominator. Only papers that met the 2003 ABPA in CF consensus criteria [7] and provided appropriate denominators were included.

### Annual data reports

We accessed the most recent annual reports published from those countries with National CF registries. Only reports that presented the number of CF patients stratified by age were used. Some of these reports also included the number of patients who had been diagnosed with ABPA, however very few stratified this finding by age.

We gained information regarding the number of individuals with CF from 2011 annual data reports for the UK [9], US [20], France [21], Germany [11], Canada [22], Australia [23], Ireland [10], the Netherlands [12] and New Zealand [24]. In addition 2010 annual data reports from Brazil [25] and Belgium [26] were also used. The locally diagnosed figures for ABPA are shown to provide a comparison with our estimates to indicate the possibility of underdiagnoses.

### Registry data

The European CF Society (ECFS) provided additional patient registry data, expanding on their latest 2008–2009 ECFSPR report [27], [28]. The ECFS includes data for Austria, Belgium, Czech Republic, Denmark, France, Germany, Greece, Hungary, Ireland, Israel, Italy, Latvia, The Netherlands, Portugal, Republic of Moldova, Serbia, Slovenia, Spain, Sweden and Switzerland [27], [28].

We contacted representatives from all CF registries we could identify by email to ask for more specific data from each country. We requested information for the number of patients with CF, the age stratification of these patients- preferably in 5 year age blocks, the sex of these individuals and the number diagnosed with ABPA alongside their diagnostic criteria for this. Specific data requests were made in the UK, the Netherlands, Belgium, Latvia, US and Ireland. These representatives provided their data directly (personal communications). Many other contacted representatives explained that the ECFS annual report was the best way to get their information. Unfortunately some countries did not have representatives to email and some did not reply.

For countries without a national registry, data were scant and not included. We did not attempt to access information from countries or continents with a low Caucasian population such as Asia or Africa. If we gained two sets of information for a country we used the most recent and complete set.

### Estimation method

We have used the percentage of individuals within each latent class from the work by Baxter et al [19]. This study only included individuals over the age of 18 therefore we are only representing the adult population for the various countries. We have extrapolated these percentages to show the estimated number of individuals in each category and added 95% confidence intervals to these percentages to provide a limited form of sensitivity analysis in the absence of other data on which to base our estimations. The figures we have used are shown in Table 1 below. Where country registry data was incomplete, but an estimate of the proportion of data is known, we have increased the estimates according to that proportion.

**Table 1:** Percentages of patients in different classes of aspergillosis with 95% confidence intervals [19].

	Number of patients	Percentage (%)	95% Confidence Interval (%–%)
No aspergillosis	49	37.7	29.8–46.3
ABPA	23	17.7	12.1–25.2
*Aspergillus* sensitisation	19	14.6	9.6–21.7
*Aspergillus* Bronchitis	39	30.0	22.8–38.4
Total	130	100.0	

### Age distribution of ABPA documented

**Figure 1:**
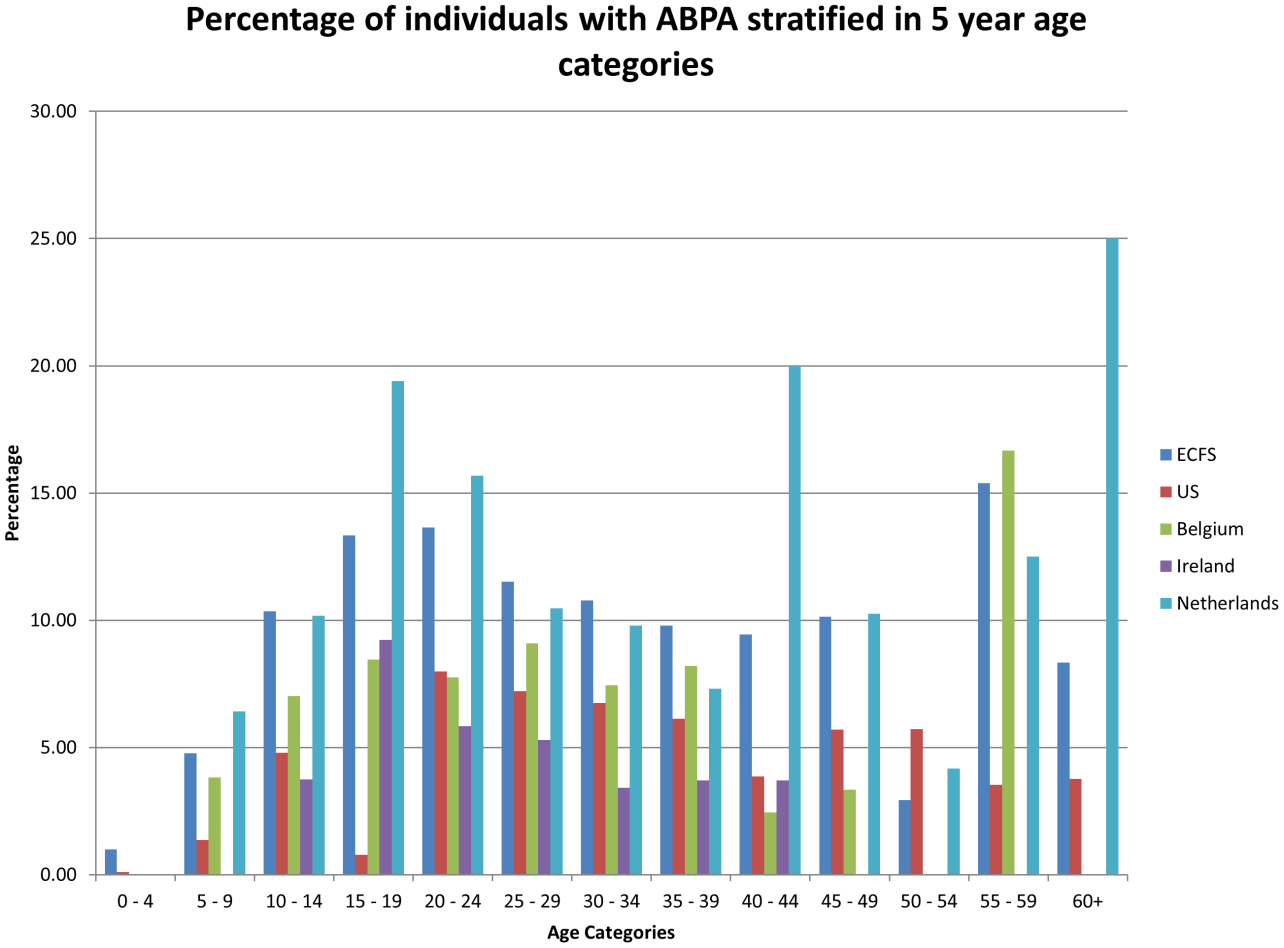
The percentage of individuals with CF stratified in 5 year age categories for numerous countries.

We compared the age distribution of ABPA for the countries that had their data stratified into 5 year age blocks (ECFSPR data, US, Belgium, Ireland, and the Netherlands) (Figure 1). We used more up to date data than in the ECFSPR report for Belgium and Ireland that we gained directly from their national registries.

## Results

We accessed sufficient data to estimate the prevalence of the different phenotypes of aspergillosis in 30 countries (Table 2). The ECFS provided data for Austria, Belgium, Switzerland, Czech Republic, Germany, Denmark, Spain, France, Greece, Hungary, Ireland, Israel, Italy, Latvia, Republic of Moldova, Portugal, Serbia, Sweden and Slovenia. The more recent data obtained for France, Germany, Ireland and Latvia superseded the ECFS report. We estimate 6,483 adults with ABPA compared with 1,564 (24%) recorded in registries, 10,988 with *Aspergillus* bronchitis and 5,347 who are sensitised to *Aspergillus fumigatus*.

**Table 2:** Estimated and recorded prevalence of ABPA and estimated incidence of Aspergillus bronchitis and prevalence of Aspergillus sensitisation by country.

Country	Year	Est. CF pop. (>18 yrs)	None	Asp sensitisation	Asp bronchitis	ABPA	Documented ABPA (%)
United States	2013	13657	5347	2071	4255	2510	869(34.6%)
UK	2011	5290	1994	772	1587	936	654 (69.8%)
France	2009	2873	1083	420	862	509	514 (101.2%)
Germany	2009	2782	1049	406	835	492	204 (41.5%)
Canada	2011	2238	844	327	671	396	N/A
Italy	2009	2550	961	372	765	451	N/A
Australia	2012	1556	587	227	467	275	N/A
Spain	2009	977	368	143	293	173	43 (25.1%)
Brazil	2010	367	139	54	110	65	N/A
Netherlands	2011	768	290	112	230	136	69 (50.8%)
Poland	2007	354	133	52	106	63	N/A
Belgium	2010	602	227	88	181	107	N/A
Ireland	2011	559	211	82	168	99	25 (25.3%)
Austria	2009	210	79	31	63	37	13 (34.4%)
Switzerland	2009	75	28	11	23	13	8 (62.8%)
Sweden	2009	368	139	54	110	65	2 (3.5%)
Hungary	2009	223	84	33	67	40	N/A
Israel	2009	302	114	44	91	53	21 (39.5%)
Czech Republic	2009	216	81	32	65	38	N/A
Greece	2009	195	74	28	59	35	5 (14.5%)
Denmark	2009	259	98	38	78	46	N/A
Slovakia	2007	216	81	32	65	38	N/A
New Zealand	2011	205	77	30	62	36	N/A
Norway	2011	178	67	26	53	32	N/A
Portugal	2009	79	30	11	24	14	2 (17.1%)
Serbia	2009	37	14	5	11	7	2 (32.3%)
Slovenia	2009	19	7	3	6	3	4 (121%%)
Uruguay	2010	19	7	3	6	3	N/A
Republic of Moldova	2009	6	2	1	2	1	N/A
Latvia	2013	8	3	1	2	1	0
**Estimated affected**		**37714**	**14218**	**5506**	**11314**	**6675**	**1774**

The USA is the country with the biggest CF population (27,840 patients in 2013). Of these patients 13,657 were ≥18 years old (B. Marshall, pers. Comm.). We estimated that 5,149 (95% CI: 4,070–6,323) would have no disease; 2,417 (95% CI: 1,652–3,442) would have ABPA; 1,994 (95% CI: 1,311–2,964) would have AS and 4,097 (95% CI: 3,114–5,244) would have AB. Data registrations in the US showed 869 (6.4%) individuals >18 were diagnosed with ABPA and 854 (6%) of those <18 years old with CF (n = 14,183) had ABPA. The adult estimate is a third (34.6%) of our estimated number. [29] One review found prevalence rates of ABPA in various studies from the USA have ranged from 1.9% to 10.8% [30]. A large scale study in the USA involving 14,210 patients with CF found only 2.1% of patients to have been diagnosed with ABPA [6].

The UK has 8,679 CF patients registered with 4,933 over the age of 18. As this only represented 89% of the population we provide estimates based on 5,290 adults (Port CF, pers. comm.). There were 1,994 (95% CI: 1,576–2,449) patients likely to have no aspergillosis or sensitisation, 936 (95% CI: 640–1,333) with ABPA, 772(95% CI: 508–1,148) with AS and 1587 (95% CI: 1,206–2,031) with AB. Interestingly 582 individuals had been diagnosed with ABPA >18 years of age in 2011 within the UK (Port CF, Pers. comm.) Two very low prevalence rates of ABPA have been suggested in previous research of 5.8% [31] and 6.8% [32]. These studies are slightly dated and used relatively low sample sizes. In 2008 another small scale study was carried out that diagnosed 8.75% of patients with ABPA [33]. Six adults with AB were identified in Leeds previously [34]. The number of children with ABPA in the UK is recorded as 324, (8.16% of children with CF.) Diagnosed ABPA rates in children and adolescents in the UK were 0.1% (0–3 yrs), 3.1% (4–7 yrs), 9.9% (8–11 yrs), 17.6% (12–15 yrs) and 15.7% (16–19 yrs) (Port CF, Pers. comm).

France has the third largest CF population documented (5,993 patients, 2,586>18years) [21] This only represented 90% of the total CF population in France therefore we based our results using an estimation of 2,873 adults. Our estimations placed 1,083 (95% CI: 856–1,330) patients in the no disease class, 509 (95% CI: 348–724) in the ABPA group, 420 (95% CI: 276–624) in AS group and 835 (95% CI: 634–1068) in the AB class. The 2009 ECFSPR had data for 5,640 individuals with CF, 2,586 were >18 years old and 463 had ABPA, representing 90% of an assumed total of 514 adults [28]. Therefore currently ABPA is probably fully captured by French CF physicians, a substantial improvement since 2000 which found the prevalence of ABPA in France to be just 3.9% [30]. Two recent studies apparently under-reported ABPA compared with notification: 8.9% [35] and 7.4% (95% CI: 6.5–8.4) of 2,935 CF patients had ABPA [5]. The number of children with ABPA documented in France is 277 (9.1%) [21].

Germany had 4,877 CF patients with data in 2011, with 2,504≥18 years old [11]. As this was only 90% of the CF population we used an adjusted total of 2,782 for our estimates. Our estimations suggested 1,049 (95% CI: 829–1,288) of these individuals would be in the non-disease class, 492 (95% CI: 337–701) to have ABPA, 406 (95% CI: 267–604) would be sensitised to Aspergillus and 835 (95% CI: 634–1,068) in the AB group. The annual data report suggested only 184 patients ≥18 years [11] had been diagnosed with ABPA. This is 7.3% of this adult population which is substantially lower than the 17.7% estimated by Baxter et al. Data from the Epidemiologic Registry of Cystic Fibrosis found 11.5% of 3,070 patients in Germany' population had ABPA [5]. The ECFSPR had data from 5,048 CF patients in Germany in 2009 and found 176 out of 2,619 individuals ≥18 years had ABPA; this is just 6.72% of this population which is once again dramatically lower than our estimates [28]. 117 children were documented to have ABPA in Germany, this is 4.81% of all Germany's children with CF [27].

Canada has 3,913 people affected by CF registered in 2011 with 2,234 ≥18years old [22]. Our estimation places 844 (95% CI: 667–1,036) without aspergillosis or sensitisation; 396 (95% CI: 271–564) in the ABPA group; 327 (95% CI: 215–486) with *Aspergillus* sensitisation and 671 (95% CI: 510–859) in the AB group. Geller et al. only found 1% of patients in Canada had ABPA in CF [6], however this is outdated research from 1999 and they may not have been using the correct diagnostic criteria, notably the 2003 ABPA in CF consensus criteria [7]. The Canadian annual data report only gave data regarding the number of patients with a positive culture of *Aspergillus*, which was 22% in 2011 [22]. A positive *Aspergillus* culture does not necessarily suggest an individual will have a disease related to that fungus, but is likely to represent either ABPA or AB.

Australia has 3,156 patients with CF registered with 1,556 of 18 years or more in their 2012 report [23]. Of the 1,556 adults with CF we estimate 587 (95% CI: 464–720) to be non-diseased; 275 (95% CI: 188–392) to have ABPA; 227 (95% CI: 149–338) to have AS and 467 (95% CI: 355–598) to have AB. One study found a lower ABPA prevalence rate in Australia of 4% [36]. The Australian annual data report does not include data about ABPA however they reported 25.2% of patients had *Aspergillus spp*. in their sputum in 2011 [23].

The Netherlands has 1,374 patients registered (2011 data) and 745 of these patients are 18 years or older [12]. The registered total is 97% of the total CF population so we based our estimated on 100% of the population (768 adults). Using the percentages of patients suspected in each latent class we estimate 290 (95% CI: 229–356) to have no disease; 136 (95% CI: 93–194) to have ABPA; 112 (95% CI: 74–167) to have AS and 230 (95% CI: 175–295) to have AB. The Netherlands has 67 patients over the age of 20 diagnosed with ABPA (V. Gulmans, personal communications) which is approximately 50% of the number suggested using Baxter's data. A European epidemiologic study of ABPA in CF found 6.8% (95% CI: 4.4%–9.9%) of patients in the Netherlands had ABPA [5]. The total number of CF patients documented to have ABPA was 128 (9.32%).

Belgium 2010 report documented 100% of their CF data that included 1,138 patients, 602 of these were ≥18 years old [26]. We estimate 227 (95% CI: patients to have no disease, 107 adults to have ABPA, 88 patients to have AS and 181 patients to have AB. Only 43 individuals >18 years had been diagnosed with ABPA in Belgium in 2010 (M. Thomas, pers. comm.) The ECFS 2009 had data for 1,129 individuals with CF in Belgium. 48 of the 581 individuals ≥18 years had ABPA [28]. These studies show 7.14% and 8.26% respectively would have ABPA; both are significantly lower than our estimate. Data from the ERCF found 13.6% (95% CI: 5.2–27.4) of CF patients in Belgium had ABPA [5] which is closer to our estimates. 32 (5.97%) children with ABPA were documented.

Ireland has a very high prevalence of CF. Ireland had 100% of data for CF patients with 559 of these aged 18 or older [10] from a population of 1073 patients. Of these 559 adults we estimate 211 (95% CI: 167–259) to not have disease, 99 (95% CI: 68–141) to have ABPA, 82 (95% CI: 54–121) to have AS and 168 (95% CI: 127–215) to have AB. In 2011, Ireland had 21 individuals over the age of 16 who have been diagnosed with ABPA [10] which is almost a fifth of our estimate despite including patients aged 16–18. The ERCF found 11.0% (95% CI: 8.6–13.7) to have ABPA in a population of 621 CF patients [5]. This figure is much greater than that from the annual data report and differs from the ECFSPR that found 2.32% of the 517 individuals with CF over 18 had ABPA [28]. The number of children in Ireland documented to have ABPA is just 15 (2.92%).

Data has also been published in a conference report for Hungary, Poland, Czech Republic and Slovakia for CF patients registered in these countries in 2007 [37]. This report documented information for 1,183 patients in Poland who have CF with 354 of these ≥18 years [37]. It is unknown whether this conference report involved 100% of CF patient data for Poland; therefore we used the numbers documented. We have estimated that 133 (95% CI: 105–164) have no disease; 63 (95% CI: 43–89) have ABPA; 52 (95% CI: 34–77) have AS and 106 (95% CI: 81–136) have AB. Unfortunately this report had no data for the number of these CF patients with ABPA to give a comparison. We had no other studies that estimated the number of individuals with ABPA in any of these countries.

Despite Brazil reporting 1,555 patients with CF in 2010 only 316 of these patients are 18 years or older [25]. These figures only represented 86% of the population therefore we based our results on 367 adults. We estimate 139 (95% CI: 109–170) of these adults will be classed as having no-disease, 65 (95% CI: 44–93) are in the ABPA group, 54 (95% CI: 35–80) classed with AS and 110 (95% CI: 84–141) to have AB. Brazil only had 15 individuals diagnosed with ABPA in 2010 out of the total 1,555 patients [25], again much lower than our estimate of 56 in the adult population. Amongst 74 individuals with CF in Bahia, Brazil, 2 (2.7%) were diagnosed with ABPA [38]. This study recognised the need to monitor these patients for ABPA as many were sensitised to *A. fumigatus*.

We had two sets of data for Hungary. 2007 data published in a conference report involved 576 patients with 238 of these ≥18 years old [37]. The ECFSPR register contains data for 555 CF patients from 2009, 201 of whom are ≥18 years old[27]. We used 223 adults to make our estimations as only 90% of the CF population was documented. We estimated 84 (95% CI: 67–103) would have no disease; 40 (95% CI: 27–56) would have ABPA; 33 (95% CI: 21–48) AS and 67 (95% CI: 51–86) would have AB. The ECFSPR did not have any data regarding the number of individuals currently diagnosed with ABPA [27].

Slovakia had similar figures to Hungary in the 2007 report with 416 individuals with CF, 216 who are >18 years old [37]. Of these 216 we estimated 81 (95% CI: 64–100) would have no disease; 38 (95% CI: 26–54) would have ABPA; 32 (95% CI: 21–47) would have AS and 65 (95% CI: 49–83) would have AB. There was no other research showing the number of people with ABPA in Slovakia.

New Zealand reported 100% of CF data in 2011 with 415 patients reported to have CF. There were 205 patients over 18 years of age [24]. We estimated that 77 (95% CI: 61–95) of these would have no disease; 36 (95% CI: 25–52) would have ABPA; 30 (95% CI: 20–44) would have AS and 62 (95% CI: 47–79) would have AB. The New Zealand 2011 annual data report gave details of the number of patients with a positive culture of *Aspergillus* age stratified in 5 year age blocks [24]. They found 22.1% of patients >16 years old were positive for *Aspergillus* [24]. A small study involving 277 Australasian (Australia and New Zealand) CF patients reported that 4% were diagnosed with ABPA in 2002 (criteria for diagnosis were in line with the 2003 criteria [7]) and 18.8% were colonised or infected with *Aspergillus* [36].

We gathered data for the Czech Republic from the 2007 conference report which involved 490 patients with CF and 190 adult CF patients (≥18 years old) [37]. The ECFSPR also had more recent data for the Czech Republic with 507 CF patients in 2009, 216 of which were ≥18 years [27]. 100% of CF patients data was documented and we therefore estimated that 81 (95% CI: 64–100) would have no disease; 38 (95% CI: 26–54) would have ABPA; 32 (95% CI: 21–47) would have AS and 65 (95% CI: 49–83) would have AB. The ECFSPR found that 10 people over the age of 18 were diagnosed with ABPA, however data was missing for 22 adults [27]. 16 children were documented to have ABPA; this is 5.5% of the total number of children with CF.

The Norway CF patient registry involved 100% of their CF patients data and shows 178 patients over 18 years in 2011 [39]. According to our estimates 67 (95% CI: 53–82) of these patients would have no disease; 32 (95% CI: 22–45) would have ABPA; 26 (95% CI: 17–39) would have AS and 53 (95% CI: 41–68) would have AB. Unfortunately this registry does not contain data regarding the number of individuals with ABPA.

The CF registry in Uruguay had 86 patients recorded with 19 over the age of 18 [40]. We were unsure as to the percentage of patients represented however using these figures we estimated 7 (95% CI: 6–9) would have no disease; 3 (95% CI: 2–5) would have ABPA; 3 (95% CI: 2–4) would have AS and 6 (95% CI: 4–7) would have AB. Once again no data was available for the number of CF patients with ABPA. The state social security institution in Uruguay (BPS) was giving treatment and following up 170 patients in 2012. They estimate that there are another 150 patients not diagnosed with CF in Uruguay. They suggest 29% of patients are over 18 years of age; therefore approximately 49 of those diagnosed patients would be adults. This suggests the CF registry in Uruguay underestimates the number of patients in their country with CF, therefore our aspergillosis estimates may be too low[41].

Finally we analysed the 2013 data sent from Latvia involving 38 patients, 8 of whom are older than 18 (K. Malina, pers. comm.) Of these 1 would have ABPA and 1 AS and 2 would have AB. No patients in Latvia are currently diagnosed with ABPA in the ECFR [5] or ECFSPR datasets (n = 28) [27].

The ECFSPR provided information for Spain. They had 740 patients registered with CF in 2009 and 293 were ≥18 years [27]. Only 30% of Spanish CF patients were included in the ECFSPR therefore we based our results on 977 adults (100%). We estimate that 368 (95% CI: 291–452) of these patients would have no disease; 173 (95% CI: 118–246) would have ABPA; 143 (95% CI: 94–212) would have AS and 293 (95% CI: 223–375) would have AB. Spain has only identified 13 individuals with ABPA over the age of 18 (4.44%); this is dramatically below our estimates. 12 (2.69%) children were documented to have ABPA [27]. Registro Europeo de FQ published data for Spain between 2008–2010 that noted 20 (3.2%) patients of all ages to have ABPA [42]. Another paper looking at the serious fungal infections that occur in Spain estimated 59,210 ABPA patients with asthma as their underlying diagnosis, a rate of 126 individuals per 100,000, although it not known how many are actually diagnosed [43].

The ECFSPR from 2009 had approximately 87.5% of the CF Swedish population recorded. There were 578 patients and 322 were 18 years or older [27]. Our estimations, based on 100% population (368 adults) found 139 (95% CI: 110–170) would have no disease; 65 (95% CI: 45–93) would have ABPA; 54 (95% CI: 35–80) would have AS and 110 (95% CI: 84–141) would have AB. Sweden only documented 2 people ≥18 years with ABPA (0.62%), however they did have 6<18 years with ABPA, this is 2.34% of children with CF [27]. This shows that it would be beneficial to extend this research to include both children and adults. Mastella et al. diagnosed ABPA in 2.1% out of 424 individuals of all ages with CF in 2000 [5].

Only 14% of CF patients' data are recorded by the ECFSPR for Italy. Of the 357 patients ≥ to 18 years [27] we estimated that if 100% of patients were documented there would be 2,550 adults. Using 2,550 we estimated 961 (95% CI:760–1181) of these patients would have no disease; 451 (95% CI: 309–643) would have ABPA; 372 (95% CI: 245–553) would have AS and 765 (95% CI:581–979) would have AB. Italy has no data published regarding ABPA prevalence; this may be partly due to the lack of a National Registry within this country.

All centres of Israel participate in the ECFSPR giving data for approximately 90% of the patients with CF. In this report there are 533 patients with CF, 272 are ≥18years of age [27]. We predicted that if 100% of data were present there would be 302 adult patients. Therefore we estimated: 114 (95% CI: 90–140) of these patients would have no disease; 53 (95% CI: 37–76) would have ABPA; 44 (95% CI: 29–66) would have AS and 91 (95% CI: 69–116) would have AB. Israel had reported 19 patients ≥18 to have ABPA in 2009 [27]. This is approximately 7% of the adult CF population, once again much lower than our estimation. Israel has 14 (5.36%) children documented with ABPA.

Denmark has a national CF registry with 100% of patients participating in the ECFSPR. In 2009 there were 451 patients registered, 259 of these patients were adults and included in our estimate [27]. We predicted 98 (95% CI: 77–120) of these patients would have no disease; 46 (95% CI: 31–65) would have ABPA; 38 (95% CI: 25–56) would have AS and 78 (95% CI: 59–99) would have AB. Denmark had no information registered regarding the number of patients with ABPA in the ECFSPR[27]. However some research was published in 2000 showing that 3.4% of patients in a sample of 353 patients with CF had CF [5]. This is dramatically below our estimated value; however the research is relatively dated and occurred prior to more modern diagnostic techniques and consensus criteria.

The ECFSPR had data for approximately 34% of the Austrian population with CF. There were 352 patients in the sample and 82 of these patients were adults therefore we estimated that in a 100% sample there would be 210 adults. Using this figure we found 79 (95% CI: 63–97) of these patients would have no disease; 37(95% CI: 25–53) would have ABPA; 31 (95% CI: 20–46) would have AS and 63 (95% CI: 48–81) would have AB The ECFSPR data described 5 people (6.1%) with ABPA [27]. Another epidemiology study of ABPA in CF in several countries found that 5.3% of 169 patients with CF in Austria had ABPA. [5] There were 6 individuals <18 years (2.22%) that were documented to have ABPA [27].

Switzerland also has a low percentage of CF patients in the ECFSPR with just 24% recorded (190 patients). Only 18 of these patients are in the adult population [27], however when adjusted to 100% there were 75 adults. We estimated 28 (95% CI: 22–35) of these patients would have no disease; 13 (95% CI: 9–19) would have ABPA; 11 (95% CI: 7–16) would have AS and 23 (95% CI: 17–29) would have AB. Two of the 18 adults (11%) were diagnosed with ABPA in the ECFSPR report. However 32 children were diagnosed with ABPA, this is 18.6% of the 172 children with CF [27]. We believe this data is incomplete as the number of patients in the >18 years category appears far too low.

Serbia had 122 patients recorded in the ECFSPR, which is approximately 95% of the total number of patients with CF. Serbia has a relatively low population of adults with CF, just 35 [27], 37 when adjusted to 100% providing estimates of 7 with ABPA, 5 with AS and 11 with AB. Two adults with ABPA were mentioned in the ECFSPR dataset. Only 1 child was documented to have ABPA [27].

Portugal does not have a national registry and therefore approximately 42% of data for CF patients in Portugal are within the ECFSPR report. Of the 117 patients with data 33 are 18 years or older [27] which we have estimated as 79 adults if 100% of data was present. We estimated that 30 (95% CI: 23–36) of these patients would have no disease; 14 (95% CI: 10–20) would have ABPA; 11 (95% CI: 8–17) would have AS and 24 (95% CI: 18–30) would have AB. Only 1 patient was reported to have ABPA in ECFSPR [27].

Greece has just 20% CF patient representation in the ECFSPR report. There are 92 patients in the registry and 39 of these are adults [27]. We estimated that 195 adults would be present if there was 100% documentation. Therefore we estimate 74 (95% CI: 58–90) of these patients would have no disease; 35 (95% CI: 24–49) would have ABPA; 28 (95% CI: 19–42) would have AS and 59 (95% CI: 44–75) would have AB. Only 1 patient had been diagnosed with ABPA [27].

The ECFSPR has only 66 patients registered for Slovenia (75% of the Slovenia CF population.) Fourteen patients are >18 years [27] (19 if 100% of data). Of these 19 patients, we estimated that 7 would have no disease; 3 would have ABPA; 3 would have AS and 6 would have AB. No patients in the adult population are diagnosed with ABPA. However 4 children were diagnosed with ABPA, this is 7.69% of the 52 children [27].

Finally the ECFSPR captured 100% of data for the Republic of Moldova CF patients, with only 41 patients [27]. Using the 6 adult patients within this cohort we calculated that one would have ABPA, one would have AS and 2 would have AB. There is currently no data available for the true number of patients who have been diagnosed with ABPA in the Republic of Moldova.

It was suggested that CF in the Indian diaspora in the UK may be in the 1 in 40,000 to 1 in 10,000 range [44]. In addition using the prevalence of F508del it has been suggested there may be 10,247 to 23,730 CF patients in India [45]. Unfortunately we were unable to involve India in our results as the estimates of the total number of patients are vague and there no figures for the number of adults (if any) with CF. In 1971 the first 3 cases of ABPA were reported in India [46]. The prevalence of ABPA in adults asthmatics in India may be substantially higher than in the rest of the world which has studied these rates [14] a substantial 27.2% in 564 adult asthmatics in North India [47].

We compared the number of individuals with CF who were <18 years and >18 years. This data provides insight into the success of care in each country as a larger proportion of their CF population >18 years would suggest better quality of care. The results are shown in Table S1 and Figure S1 which rank the countries from the highest to the lowest percentage of >18 years. We have not included New Zealand in this figure as we only have data for the number <16 and >16 years, or Switzerland because these data are notably incomplete.

The ECFSPR recorded the number of individuals across Europe with ABPA in 5 year intervals (figure 1). Out of 17,737 individuals, 2,203 had unknown/missing data, 14,244 had no ABPA in the year of follow up and 1,290 had ABPA [27]. Individuals aged 0–4 and 5–9 had a very low percentage of individuals with ABPA of 0.91% and 4.11% respectively. The percent with ABPA increased to 8.51% of 10–14 year olds and plateaued at ∼10.3% of 15–19 and 20–24 year olds. Of the 1,290 individuals with ABPA, 1,024 (79%) were between the ages of 10 and 34. After 34 years, the number of people with CF in Europe decreases and the percentages of individuals with ABPA is generally around 7% [27], perhaps indicating earlier death (or transplantation). As these data are derived from a very large population it is clear that CF patients aged 15 to 24 are most at risk of ABPA, despite the likely problems of under diagnosis.

We compared this ECFSPR with data from US, Belgium, Ireland and the Netherlands (Figure 1). Similar trends were found, such as the number of children with ABPA was <1% under 4 years and the percentages tended to increase in adolescent years and those in their 20s. The high percentage of ABPA in Netherlands in those older than 60 reflects a small number of individuals with CF in that age range. Exact data for the number of individuals with CF in each age band and the number diagnosed with ABPA are shown in Table S2. As we have not modelled aspergillosis rates in people under the age of 18 further research involving all age categories and the different classes of aspergillosis would be beneficial.

## Discussion

In this study of the majority of the world's CF adult population, we estimate there to be 6,675 adults with ABPA, compared with 2,221 (33%) recorded in registries, 11,314 with *Aspergillus* bronchitis and 5,506 who are sensitised to *Aspergillus fumigatus*. Our estimations suggest many countries need to revise their methods of screening for and diagnosing ABPA and Aspergillus bronchitis which together may affect nearly 50% of patients. We argue that the ABPA estimates have real validity because of the match with recorded cases in France. We have provided 95% confidence intervals as a limited form of sensitivity analyses, in the absence of alternative data on which to base estimations. It is highly likely that even with excellent diagnostics applied systematically across whole cohorts, that there will be variations, perhaps substantial variations, from these estimates. Variations could arise for genetic, environmental, seasonality, clinical practice, patients switching between aspergillosis phenotypes and other undefined reasons. The ranges of estimates provided for each country may cover most of the variation, but until national studies are done, this will not be known. Studies are required in many countries to determine the prevalence rates of ABPA, AS and AB using these new diagnostic methods. These confirmatory epidemiological studies could be extremely beneficial to improving the management of patients with CF across the world.

Clinically, ABPA is difficult to recognise due to its similar clinical presentation to CF exacerbations related to many pathogens. Wheezing, exercise intolerance, change in pulmonary function and increased sputum production are not specific to ABPA. Serology (notably *Aspergillus* specific IgE and *Aspergillus* IgG) and/or detection of high quantities of *A. fumigatus* in the airways are required to establish the diagnosis. In 2003 Stevens et al proposed the minimum criteria required for diagnosis. These criteria consisted of clinical deterioration; total serum IgE concentrations >500 IU/ml; immediate cutaneous reactivity to *Aspergillus* in a skin prick test; and another serological marker or chest radiography or CT scan showing recent abnormalities [7]. Our work suggests that these criteria should be revised, but the principle remains that screening for ABPA is important.

An *Aspergillus*-specific IgE (sIgE) >5.7 KUA/L has 100% sensitivity and 94% specificity to distinguish ABPA from all over forms of aspergillosis and normal [19]. Values of sIgE >3.75 KUA/L and <5.7 KUA/L denote *Aspergillus* sensitisation without ABPA (sensitivity 94% and specificity 100%). While replication of these data and cut-offs is required in another similar study, these values can be used to confirm or refute our estimates in other adult CF populations.

*Aspergillus* bronchitis is a newly recognised entity in CF [48] and as such the high proportion of patients with this condition was and remains a surprise. A two year reduction in FEV1 associated with *Aspergillus* bronchitis, which may or may not be reversible with antifungal therapy (possibly combined with targeted antibiotic therapy) is clearly a new target for intervention in CF with a reasonable prospect of significant clinical improvement. We have demonstrated excellent and partial responses to antifungal therapy in patients with Aspergillus bronchitis without CF [49]. Some patients with ABPA also have high Aspergillus IgG titres, one of the cardinal features of *Aspergillus* bronchitis in CF, and these ABPA patients may also have *Aspergillus* bronchitis. We haven't modelled this.

It is not clear currently that each Aspergillus phenotype is completely discrete. Experience alongside our data indicates that patients may switch phenotypes (19) and some may have overlap or two syndromes. For example, patients with extensive bronchiectasis and ABPA may also have AB. This needs further evaluation, and in particular non-overlapping diagnostic tests. Therefore the estimates here can only be approximate and represent a cross-sectional analysis, not a longitudinal one.

ABPA often manifests in late childhood in CF, as amply demonstrated by the registry data. Overall 984 children with ABPA were documented with rates in the paediatric population varying from a low of <1% in Serbia to 9.1% (France), 9.3% (Netherlands) and 18.6% (Switzerland). It is likely that ABPA is underdiagnosed in most countries, but diagnostic criteria in children are barely studied and so the variation could reflect differing criteria for defining cases.

Acute exacerbations of ABPA are treated with corticosteroids which are partially effective but carry a substantial penalty in CF of more frequent diabetes and osteoporosis, both significant clinical problems in CF. Antifungal therapy with itraconazole is only occasionally effective because of poor bioavailability in many, despite elevated doses and use of the oral solution. Voriconazole is more reliably bioavailable, but is often not tolerated and has long term adverse events such as photosensitivity, skin cancer, periostitis due to excess fluoride and peripheral neuropathy. Furthermore *A. fumigatus* has recently shown increasing resistance to these triazoles [50], [51] [52]. Appropriate use and therapeutic drug monitoring is therefore essential for these agents in CF. But assuming good exposure, the potential impact for patients with ABPA and possibly for Aspergillus is large.

Aspergillus sensitisation is of uncertain significance. It has been shown to be associated with reduced FEV_1_ in individuals with CF [53]–[55], but it is not clear if this is a causal relationship or an epiphenomenon. It is also not clear if it is amenable to antifungal therapy or any immunomodulatory treatment, or whether reduced FEV1 would be reversible or progressive loss reduced. It is also not clear if it is a precursor to ABPA or a separate distinct aspergillosis phenotype within CF.

**Figure 2:**
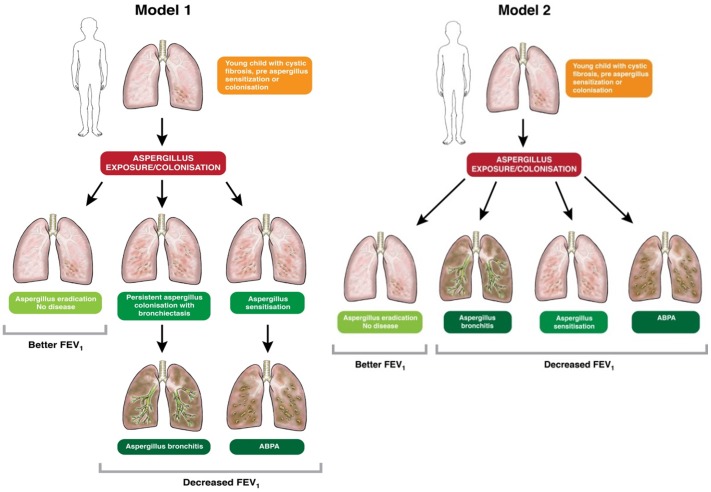
Alternative models of the development of ABPA, *Aspergillus* sensitisation or *Aspergillus* bronchitis.

We postulate there are two alternative models of the development of ABPA, *Aspergillus* sensitisation or *Aspergillus* bronchitis (Figure 2). Model 1 depicts the sequential development of ABPA after *Aspergillus* sensitisation [56] and *Aspergillus* bronchitis after the development of bronchiectasis and persistent colonisation. Model 2 depicts alternative pathways, perhaps genetically determined, without sequential development of different manifestations. This model suggests the four different disease patterns are not interdependent.

**Figure 3:**
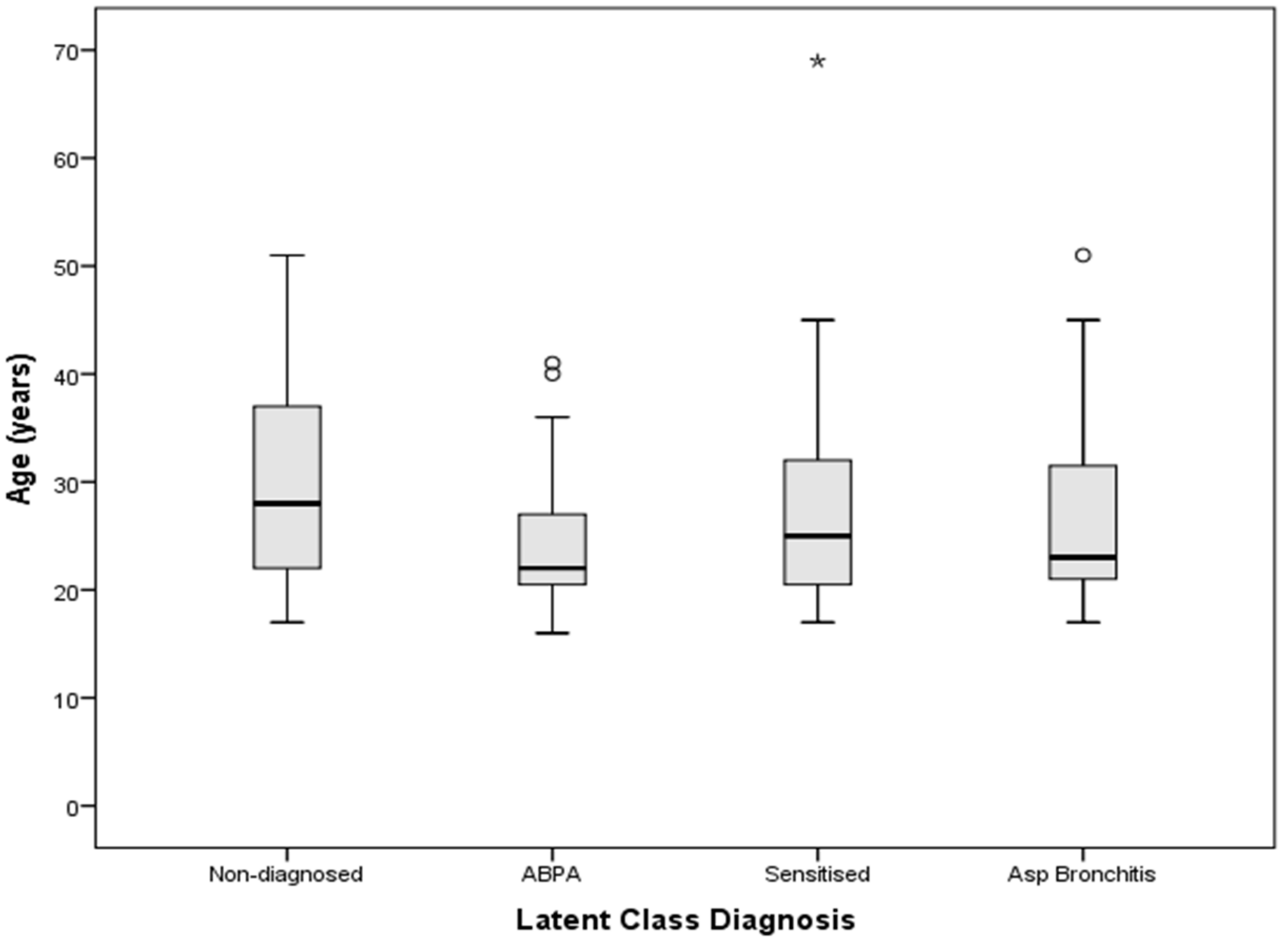
Boxplot to show the difference in ages between the different diagnostic groups of aspergillosis.

We created boxplots using the Baxter et al data and found no significant different in the ages of the individuals in the latent classes (figure 3). This finding is consistent with model 2 (Figure 2), although 17% of patients re-evaluated over a 9 month period moved between classes during follow-up. Clearly this area requires more longitudinal studies.

We have based our estimates on Model 2. The median age of the ABPA group was younger than that of the AS group, 22 years and 28 years respectively. If model 1 was proven to be correct the estimated national burdens for each class may differ. Another small scale study involving 80 paediatric patients also found the ABPA population to be younger than those who were *Aspergillus* sensitised, again making model 1 highly unlikely [33]. Hutcheson et al carried out a 12 year longitudinal study using 118 patients from a CF clinic at Cardinal Glennon Children's Hospital in St. Louis. Throughout this time they studied the development of 5 *in vivo* and *in vitro* immune parameters of *A. fumigatus* sensitisation with the aim of determining the parameters which could predict those patients who were likely to be diagnosed with ABPA in the future. They found no link between those with *Aspergillus* sensitisation and those who developed ABPA and in many cases found there was often a reduction in the positive parameters over time [57]. This finding was supported by another longitudinal study in 147 patients aged 5 to 43 years. They also found fluctuations in the levels of IgE and IgG specific to *Aspergillus* and a marked decline in total IgE over time despite ABPA status [58]. This once again suggests there is no link between AS and ABPA and therefore supports Model 2 that we have used in our estimations.

Unfortunately many countries do not have complete data sets for the number of patients with CF. This may be due to several reasons, such as the lack of a national registry; poor diagnosis of CF or many countries with a low population of Caucasians have a low prevalence rate of CF leading to late recognition. We were unable to identify any population data for countries in Africa, Asia, the Middle East, the Arctic Region or Latin America, with the exception of Brazil and Uruguay. In addition we were regrettably unable to obtain data for the following European countries: Bulgaria, Croatia, Cyprus, Estonia, Finland, Lithuania, Luxembourg, Malta, Romania, Iceland, Montenegro, Turkey, Albania, Bosnia and Herzegovina and Kosova.

Some countries without national registries did not have full data sets within the European Cystic Fibrosis Society report of 2008–2009. The 2009 estimated coverage for Austria is 39%, for Greece is 20%, Italy 14%, Portugal 42%, Slovenia 75%, Spain 30%, 85–90% in Sweden and 24% in Switzerland. We have compensated for these by assuming that the unrecorded patients are similar to those that are recorded, which may be a fallacious assumption.

The aspergillosis cases that are recorded in the registries were varied. Some just reported the number of patients with any species of *Aspergillus* detected in sputum samples (Australia [23], Canada [22], New Zealand [24]) which is likely to represent ABPA or AB, but may represent occasional colonisation. Germany's data annual report presented the number of individuals diagnosed with ABPA for both <18 and >18 [11] and the UK and Irish data shows the number of individuals >16 years old with ABPA [9], [10]. In addition following a data request the ECFS, the Netherlands, US, UK, Belgium and Latvia sent their data regarding the number of individuals with ABPA stratified in 5 year age groups. Belgium [26] and Brazil [25] only documented the total number of individuals with ABPA without any age stratification. France released data regarding the number of individuals treated for aspergillosis in 5 year age categories [21]. Some countries did not release any data regarding *Aspergillus* or ABPA such as the US [20], Poland [37], Uruguay [40], Slovakia [37], Norway [27], [39], Czech Republic [27], [37] and Hungary [37].

A key issue for the future is whether ABPA, AB and AS could be prevented. *Aspergillus* species are ubiquitous moulds found in organic debris and are common indoor moulds especially in areas with lots of dust for example the curtains, floor mats and the attic [59]. There is some evidence to show that the prevalence of ABPA increases with increasing concentrations of *A. fumigatus*, for example there is more ABPA in agricultural conditions [60] and where there is *A. fumigatus* in the immediate outside environment [61]. Manifestations of ABPA are a complex interaction between the patient's immune response and the number and virulence of the organisms [62]. It is highly likely that ABPA is a multi-genic disorder, given multiple genetic associations with ABPA [56] and so complete avoidance is unlikely to be a successful prevention strategy. Immunisation, aggressive eradication in genetically susceptible individuals and long term suppressive antifungal therapy, possibly combined with (as yet undefined) immunomodulatory therapy are likely to be the most successful prevention and management strategies for the future.

## Summary

In summary we have estimated the number of patients with ABPA, *Aspergillus* sensitisation and *Aspergillus* bronchitis in many countries based on a recent publication that used established serological techniques combined with RT-PCR and GM to reclassify aspergillosis in adult patients with CF. Further epidemiological studies are required using these new techniques to determine the prevalence of these different classes of aspergillosis within various countries globally and within the paediatric population.

## Supporting Information

Figure S1 **The percentage of cystic fibrosis patients under and over 19 years of age in numerous countries.**
(TIF)

Table S1 **The number of individuals with CF <18 and >18 years in numerous countries.**
(TIF)

Table S2 **Numbers of patients with recorded ABPA by age bands in different countries.**
(TIF)
