# Supplementary figures and images for: Multi-Country Estimate of Different Manifestations of Aspergillosis in Cystic Fibrosis
Source: PLoS One. 2014 Jun 10;9(6):e98502. doi: 10.1371/journal.pone.0098502 (PMC4051580; doi:10.1371/journal.pone.0098502)

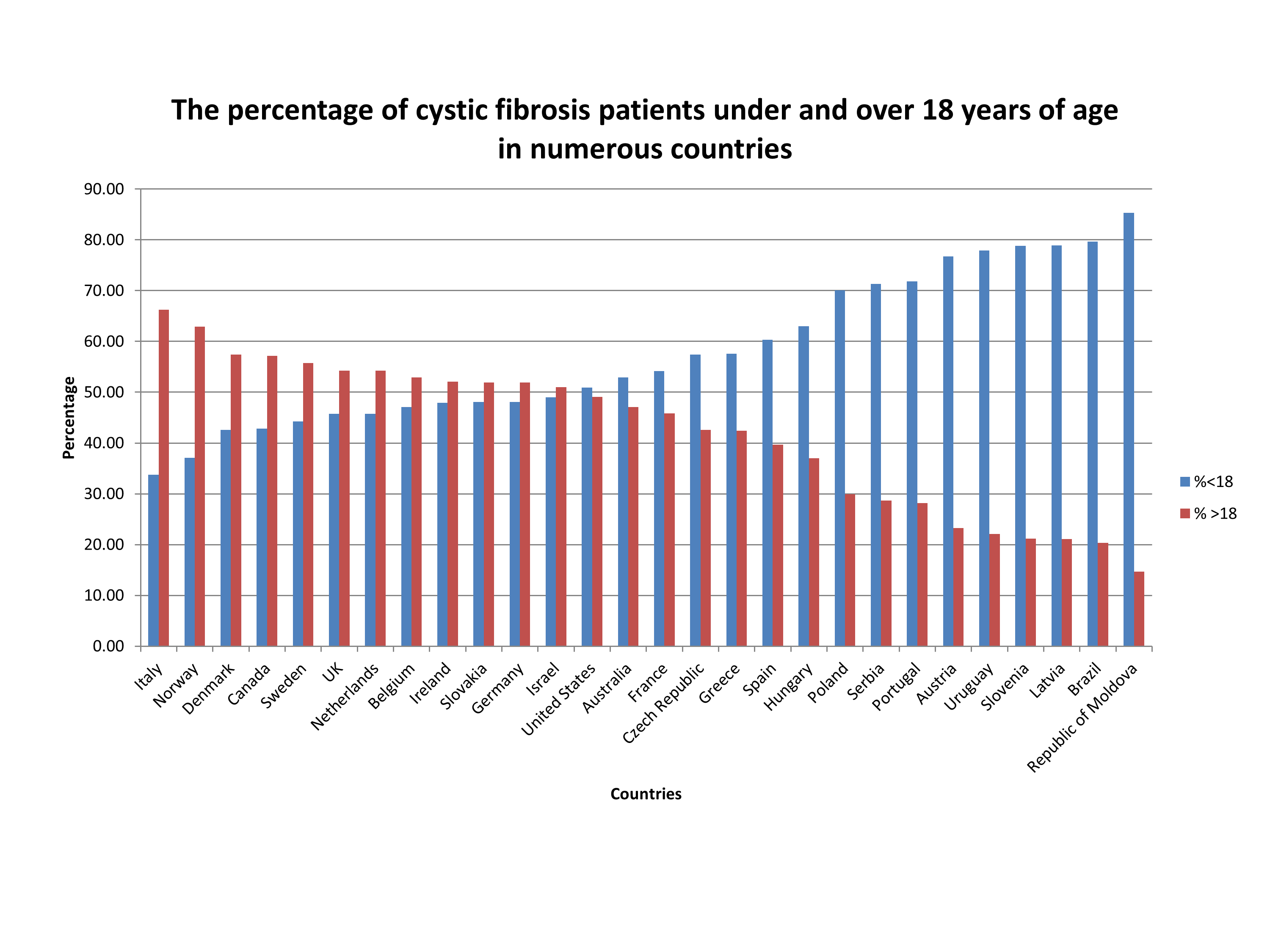

Supplement: Figure S1 — The percentage of cystic fibrosis patients under and over 19 years of age in numerous countries. (TIF) [file pone.0098502.s001.tif]

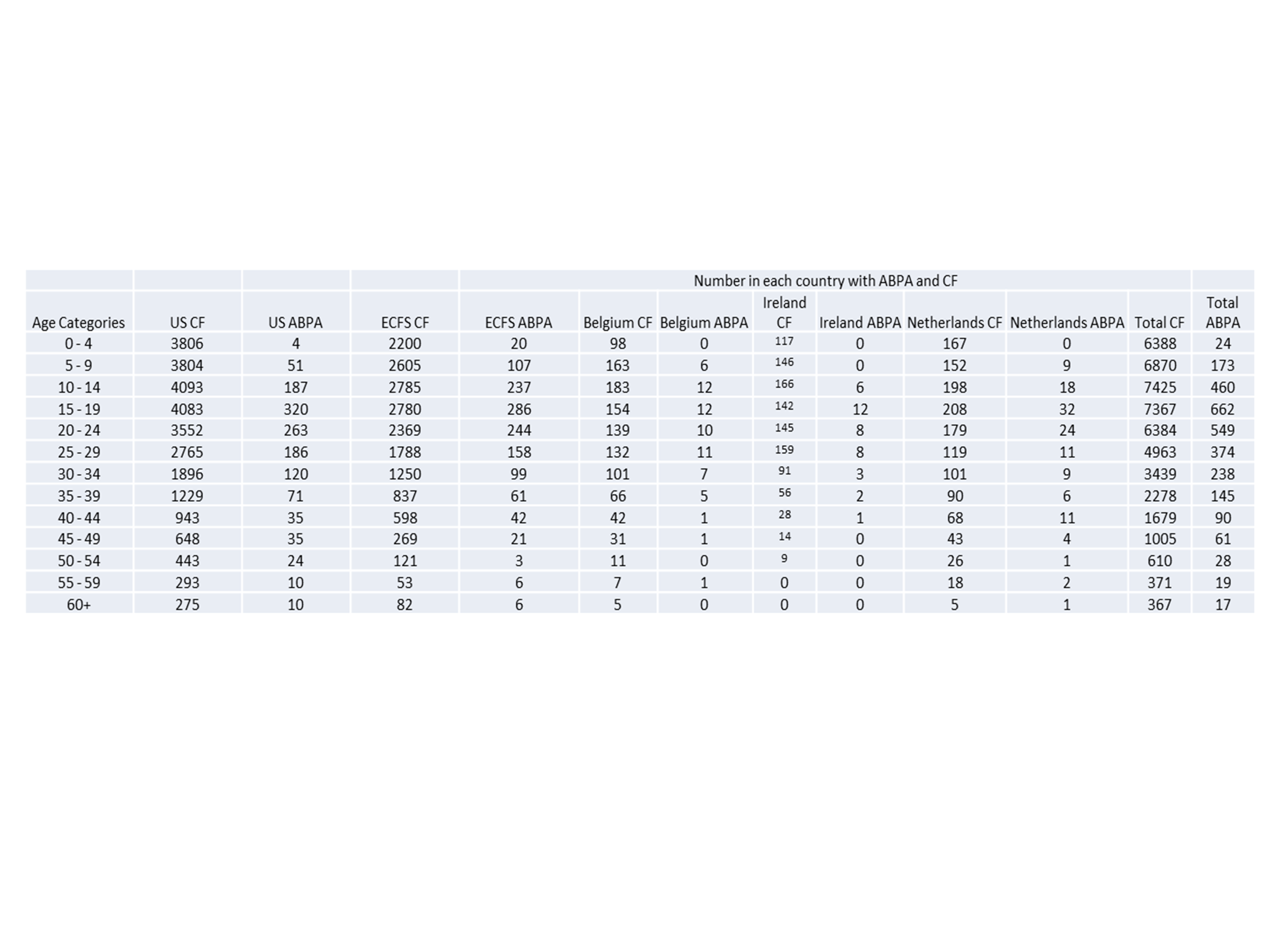

Supplement: Table S2 — Numbers of patients with recorded ABPA by age bands in different countries. (TIF) [file pone.0098502.s003.tif]
